# The Vitamin D Receptor Is a Wnt Effector that Controls Hair Follicle Differentiation and Specifies Tumor Type in Adult Epidermis

**DOI:** 10.1371/journal.pone.0001483

**Published:** 2008-01-23

**Authors:** Héctor G. Pálmer, Fernando Anjos-Afonso, Geert Carmeliet, Hikaru Takeda, Fiona M. Watt

**Affiliations:** 1 Cancer Research UK Cambridge Research Institute, Li Ka Shing Centre, Cambridge, United Kingdom; 2 Cancer Research UK London Research Institute, London, United Kingdom; 3 Laboratory for Experimental Medicine and Endocrinology, Gasthuisberg, Katholieke Universiteit Leuven, Leuven, Belgium; 4 Department of Dermatology, Yamagata University School of Medicine, Yamagata, Japan; Centre de Regulacio Genomica, Spain

## Abstract

We have investigated how Wnt and vitamin D receptor signals regulate epidermal differentiation. Many epidermal genes induced by β-catenin, including the stem cell marker keratin 15, contain vitamin D response elements (VDREs) and several are induced independently of TCF/Lef. The VDR is required for β-catenin induced hair follicle formation in adult epidermis, and the vitamin D analog EB1089 synergises with β-catenin to stimulate hair differentiation. Human trichofolliculomas (hair follicle tumours) are characterized by high nuclear β-catenin and VDR, whereas infiltrative basal cell carcinomas (BCCs) have high β-catenin and low VDR levels. In mice, EB1089 prevents β-catenin induced trichofolliculomas, while in the absence of VDR β-catenin induces tumours resembling BCCs. We conclude that VDR is a TCF/Lef-independent transcriptional effector of the Wnt pathway and that vitamin D analogues have therapeutic potential in tumors with inappropriate activation of Wnt signalling.

## Introduction

Adult mammalian epidermis is maintained by stem cells that self-renew and produce progeny that differentiate along the lineages of the hair follicle (HF), sebaceous gland (SG) and interfollicular epidermis (IFE) [1], [2]. The canonical Wnt pathway controls both epidermal stem cell renewal and lineage selection [3], [4], [5]. By constructing mice with a 4-hydroxy-Tamoxifen (4OHT) inducible form of stabilized β-catenin under the control of the keratin 14 promoter (K14ΔNβ-cateninER transgenics) it is possible to control the timing, location and extent of β-catenin activation in adult epidermal stem and progenitor cells [5], [6]. Activation of β-catenin induces growth (anagen) of existing HFs and induces ectopic follicles that arise from pre-existing follicles, SGs and IFE. On prolonged activation of β-catenin, hair follicle tumours are induced, consistent with the finding that human pilomatricomas have activating β-catenin mutations [6], [7], [8].

β-catenin interactions with Lef1 and Tcf3 in the epidermis have been extensively characterised [4], [9]. However, in addition, β-catenin is known to bind and activate the vitamin D receptor (VDR) [10], [11]. The VDR, like β-catenin, is essential for adult epidermal homeostasis [12], [13]. Natural mutations in the VDR gene in humans result in familial 1,25-dihydroxyvitamin D-resistant rickets (HVDRR), which can be associated with alopecia [14]. VDR null mice develop rickets and fail to undergo the first postnatal hair growth phase (anagen), resulting in alopecia and conversion of follicles into cysts with IFE differentiation [15], which is highly reminiscent of the effects of impaired epidermal Wnt signalling [16].

There is evidence for both ligand dependent and independent effects of VDR. In cultured cell lines β-catenin can bind to unligated VDR, but complex formation is enhanced by vitamin D, and β-catenin stimulates vitamin D dependent transcription [10]. In vivo, expression of a mutant VDR that can bind β-catenin but not vitamin D (L233S) rescues alopecia in VDR null mice, demonstrating ligand independent functions of the VDR in skin [11], [17]. However, some vitamin D analogues, including EB1089, induce hair growth in nude mice [18]. Leucine 417 of the VDR is essential for ligand dependent binding to the COOH-terminus of β-catenin, but not to vitamin D [11]. Expression of a VDR-L417S mutant in VDR null mouse skin delays, but does not completely rescue, alopecia [17]. Taken together, these observations suggest that the ligand dependent interaction between VDR and β-catenin contributes to hair follicle maintenance.

It has recently been reported that VDR ablation leads to a gradual decrease in epidermal stem cells, consistent with postnatal hair loss [16]. Cooperative transcriptional effects of β-catenin and Lef1 are abolished in VDR null keratinocytes, and in the absence of VDR β-catenin activation does not induce the increase in proliferation characteristic of anagen entry [16]. These observations prompted us to examine the relative contributions of VDR and TCF/Lef to β-catenin induced gene expression, and to explore the effects of VDR on lineage reprogramming and tumour formation in adult epidermis. We present evidence that the VDR is a TCF/Lef independent transcriptional effector of the canonical Wnt pathway that promotes HF differentiation and modulates Wnt-induced tumour formation.

## Results

### β-catenin is a co-activator of VDR in epidermal keratinocytes

**Figure 1:**
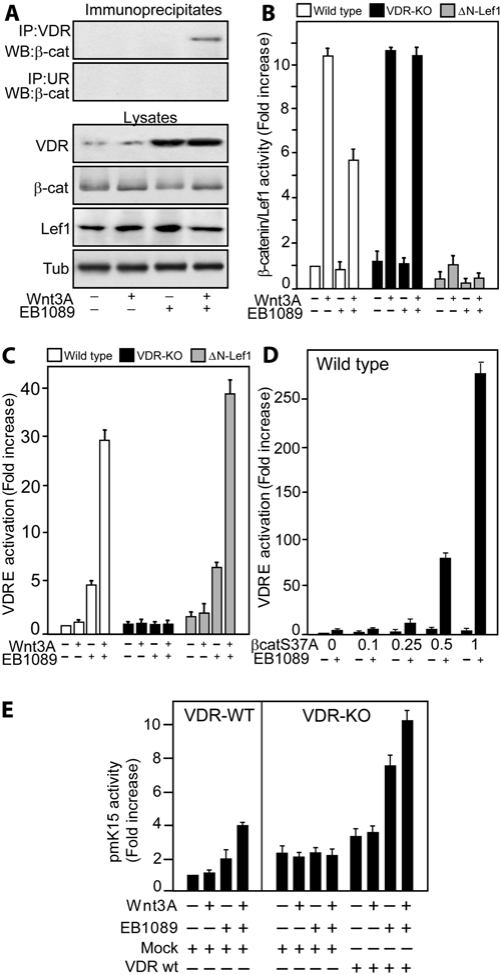
β-catenin is a ligand dependent co-activator of VDR. Primary mouse keratinocytes derived from wild type, VDR null (VDR-KO) or K14ΔNLef1 transgenic (ΔN-Lef1) mice were treated with vehicle (-), 10^−7^ M EB1089 or 100 ng/ml Wnt3A, alone or in combination. (A) Wild type cells were treated for 4 h, then lysed and immunoprecipitated with VDR or HA (unrelated; UR) antibodies. Immunoprecipitates (top two panels) or total lysates (bottom four panels) were immunoblotted with the indicated antibodies. (B) Cells were transiently transfected with TOPFlash and FOPFlash. Values correspond to the ratio of TOP/FOP. (c–f) Cells were transiently transfected with 4×VDRE luciferase reporter (C–E) or K15 promoter (F) constructs. All values were corrected for the Renilla control and are represented as fold increase relative to wild type cells treated with vehicle alone. Data are means±S.D. of triplicate samples.

Given the uncertainty regarding the role of VDR ligands in hair follicle maintenance, we began by examining the conditions under which VDR-β-catenin complexes formed in primary mouse epidermal keratinocytes. In immunoprecipitation/Western blot assays we could not detect complex formation in the absence of Wnt3A and EB1089, or when cells were treated with a single ligand. Complex formation was only detectable when cells were stimulated with both Wnt3A and EB1089 (Figure 1A). EB1089 treatment increased the level of VDR, consistent with its ability to inhibit VDR degradation [19].

We next examined how the canonical Wnt and vitamin D pathways interacted to regulate transcription. We transfected primary mouse keratinocytes with luciferase reporters of transcriptional activity: a TOPFlash reporter containing 3 TCF/Lef binding sites and a VDRE reporter consisting of 4 vitamin D response elements (4×VDRE) (Figure 1B–D). Wnt3A induced TCF/Lef1 transcriptional activity 10 fold in wild type and VDR null cells but not in cells that expressed an N-terminally truncated form of Lef1 (ΔNLef1) unable to bind β-catenin (Figure 1B). There was some repression of TOP-Flash activity upon EB1089 treatment alone, as reported previously [10], [11]. We hypothesise that this happens when nuclear β-catenin levels are limiting and the VDR and Lef/TCF proteins compete for β-catenin binding; in the presence of EB1089 more β-catenin would bind VDR and less to Lef/TCF proteins.

Wnt3A strongly enhanced induction of the vitamin D response construct by EB1089 in wild type and ΔNLef1 cells (Figure 1C). Transient transfection of a stabilised β-catenin mutant (S37A) stimulated vitamin D dependent transcription in a dose dependent manner in wild type cells (Figure 1D). As expected, there was no activation of the VDRE in VDR null cells treated with Wnt3A, β-catenin (S37A) or EB1089, alone or in combination (Figure 1C). Transfection of wild type VDR rescued the cooperative effects of Wnt3A or S37Aβ-catenin on vitamin D dependent transcription in VDR null cells (data not shown). Taken together, the transcription reporter assays and co-immunoprecipitation experiments establish that β-catenin binds the VDR and is a transcriptional co-activator of ligand-activated VDR in epidermal cells.

**Figure 2:**
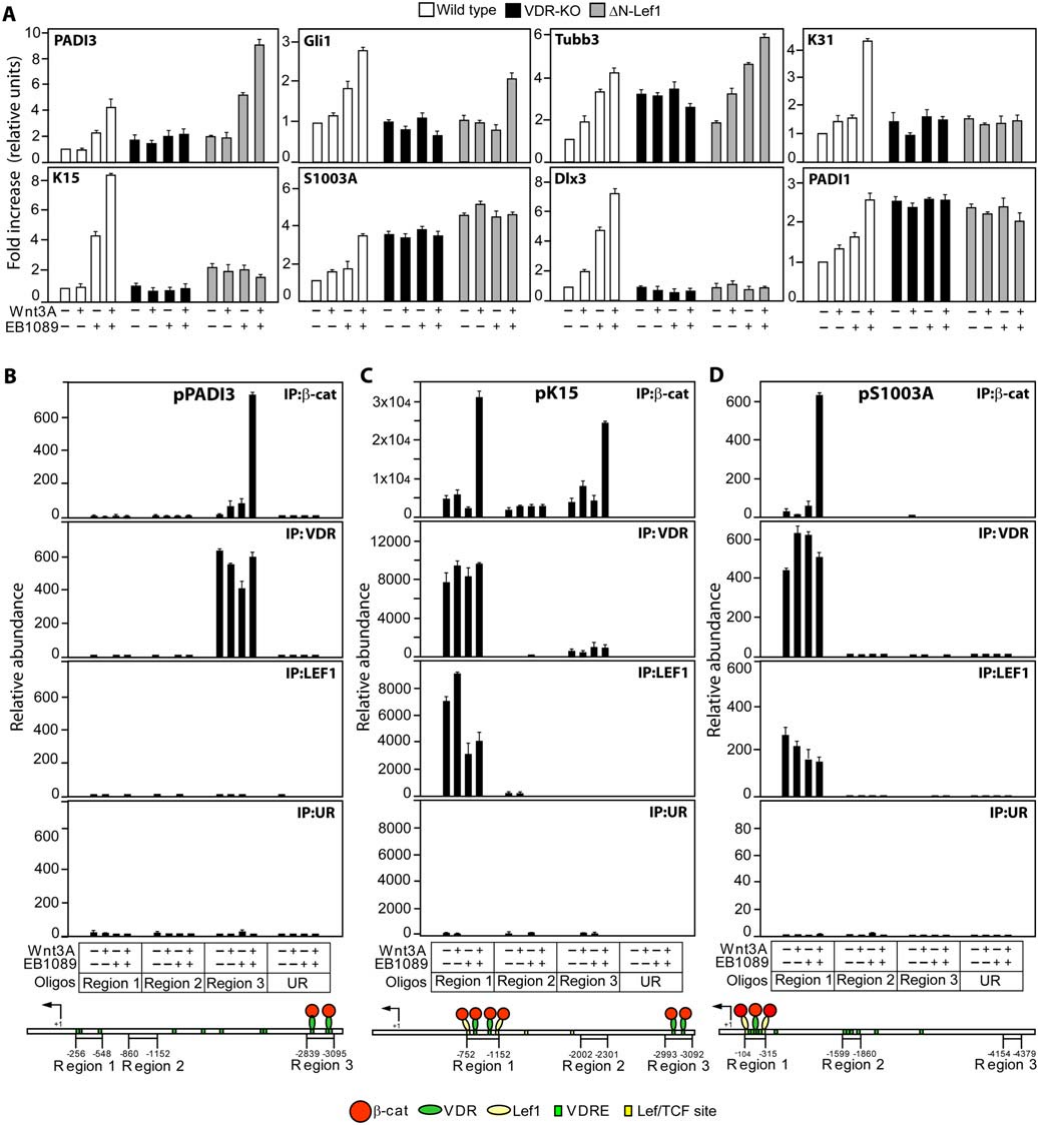
Ligand activated VDR-β-catenin complexes regulate transcription of hair follicle genes. (A–D) Cells were treated for 4 h (B–D) or 8 h (a) with (+) or without (−) Wnt3A or EB1089. (A) mRNA levels of the genes indicated were measured by real-time PCR. All values are represented as fold increase relative to wild type cells treated with vehicle alone. Data are means±S.D. of triplicate samples. (B–D) Wild type cells were lysed and immunoprecipitated with VDR, β-catenin, Lef1 or unrelated antibody (HA tag; UR). Immunoprecipitated genomic DNA fragments or input controls were amplified by real-time PCR using specific primers for three regions of the mouse promoters indicated or unrelated genomic primers (UR). Data are means±S.D. of triplicate samples. Scaled diagrams summarize location of VDREs and TCF/Lef binding sites and the proteins bound to each region in cells treated with Wnt3A and EB1089.

We next examined whether β-catenin acted as a transcriptional co-activator of VDR on a natural promoter. Keratin 15 is expressed by stem cells in the HF bulge and in β-catenin induced ectopic HF [5]. EB1089, but not Wnt3A, induced a luciferase reporter gene under the control of the mouse K15 promoter (proximal 5 kb) (Figure 1E). The highest induction occurred on combined treatment with EB1089 and Wnt3A. In VDR null cells the keratin 15 promoter was unresponsive. Transfection of an exogenous wild type VDR rescued the response to EB1089 and Wnt3A. We also performed real time PCR to investigate whether endogenous keratin 15 expression was modulated by vitamin D (Figure 2A). Wnt3A alone was unable to increase Krt15 mRNA levels in cultured wild type keratinocytes. However, EB1089 stimulated expression, and this effect was enhanced by Wnt3A in wild type but not in VDR null or ΔNLef1 cells (Figure 2A). We conclude that β-catenin acts as a transcriptional co-activator of the VDR on both artificial (Figure 1C–E) and endogenous promoters.

### VDREs exist in the promoters of β-catenin regulated genes

To investigate the significance of β-catenin/VDR interactions *in vivo*, we performed a systematic analysis of the promoter regions of genes previously reported to be upregulated by 4-hydroxy-Tamoxifen (4OHT) treatment of the skin of K14ΔNβ-cateninER transgenic mice [5]. TCF/Lef and/or VDR binding sites were significantly enriched in 91 of the 103 genes upregulated more than 3 fold at 7 days of 4OHT treatment when compared with the same number of random promoter regions in the mouse genome (Table S1, S2). Of the 91 genes only two (p, Bdh) lacked VDREs. Two thirds (61/91) had multiple VDRE and TCF/Lef sites. 15/91 had multiple VDREs and a single TCF/Lef site. Strikingly, 13 genes contained VDREs but no TCF/Lef consensus binding sites, supporting the concept that nuclear β-catenin can activate vitamin D/VDR target genes in the absence of TCF/Lef.

Many of the genes upregulated by β-catenin (Table S1) encode proteins that are specifically expressed in hair follicles (Figure S1). Using real time PCR to examine their expression in cultured mouse keratinocytes we could distinguish two categories of genes. One group, represented by PADI3, Gli1 and Tubb3, was induced by EB1089 alone or in combination with Wnt3A, in wild type and ΔNLef1 cells but not in VDR null cells (Figure 2A). We conclude that these genes are VDR dependent, TCF/Lef independent Wnt targets. The second group, comprising S1003A, Dlx3, PADI1 and Krt31, were also maximally induced in wild type keratinocytes by combined treatment with EB1089 and Wnt3A, but differed from the other category in being unresponsive in both VDR null and ΔNLef1 keratinocytes (Figure 2A). Thus these genes, like Krt15, were VDR dependent, TCF/Lef dependent Wnt targets.

In order to characterise the transcription factor complexes binding to the promoters of these genes, we performed ChIP on three of them (Figure 2B–D). We designed primers to amplify three regions in each promoter analysed, corresponding to one region with both VDREs and TCF/Lef sites, one region with VDREs alone, and one region with neither (Table S3). We used Q-PCR to determine the abundance of each region in the ChIP relative to a control, unrelated genomic region (UR). In the keratin 15 promoter β-catenin was present both in the region that contained exclusively VDREs (region 3) and in the site containing clustered VDREs and TCF/Lef sites (region 1), but only highly enriched when cells were stimulated with both EB1089 and Wnt3A (Figure 2C). Lef1 accumulated in region 1 only; VDR was most abundant in region 1 but was also detectable in region 3. In the S1003A promoter Lef, VDR and β-catenin accumulated in region 1, containing clustered VDREs and TCF/Lef sites, recruitment of β-catenin being dependent on the combination of EB1089 and Wnt3A (Figure 2D). We conclude that Krt15 and S1003A depend on β-catenin binding to ligand activated VDR and Lef1 for Wnt induction.

The PADI3 promoter differed from the S100A and K15 promoters because it had no TCF/Lef binding sites (Table S1; Figure 2B). Nevertheless, β-catenin accumulated in region 3 of the promoter, which contains three VDREs, in cells treated with EB1089 and Wnt3A. In contrast, region 1, which also has putative VDREs, was not precipitated with any antibody.

As further confirmation that β-catenin is recruited through the VDR to region 3 of the PADI3 and K15 promoters, we performed ChIP in VDR null primary mouse keratinocytes (Figure S2). Whereas β-catenin was recruited to these regions in wild type cells (Figure 2B, C; Figure S2A, B), it was absent in VDR null cells (Figure S2A, B).

We conclude that β-catenin bound and enhanced ligand activated VDR in Wnt target genes, both in presence of Lef1 (K15 and S1003A) and independently of Lef1 (PADI3).

### Ligand activated VDR cooperates with nuclear β-catenin to induce hair differentiation

**Figure 3:**
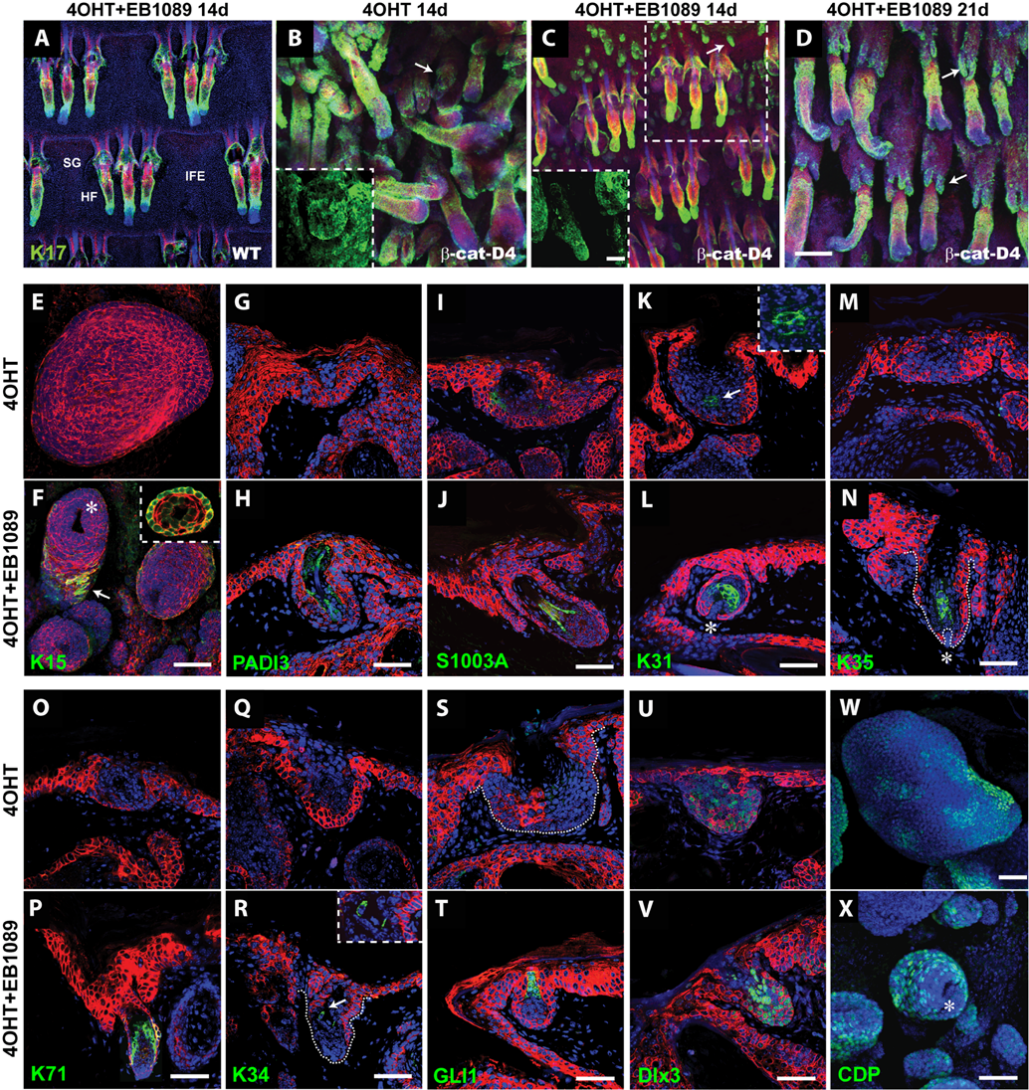
EB1089 stimulates differentiation of ectopic hair follicles formation in the D4 line of K14ΔNβ-cateninER transgenic mice. Epidermal whole mounts (A–D) or sections (E–X) of wild type (WT) or transgenic (β-cat-D4) tail skin treated with 4OHT and/or EB1089. (A–D) Immunolabelling for keratin 17 (green) with Hoechst (blue) and phalloidin-TRITC (red) counterstaining. Arrows indicate ectopic hair follicles in the IFE (B, C) or SG (D). Inserts in (B, C) show higher magnification views of ectopic follicles. (E–X) Immunolabelling with the antibodies shown (green) in combination with Hoechst (blue; E–X), phalloidin-TRICT (red; E, F) or anti-keratin 14 (red; g-v). (C) Dashed square indicates an epidermal unit as previously described [5]. (F) Arrow indicates keratin 15 expression in ORS of ectopic HF. Insert shows base of an ectopic follicle. (K) Cells expressing K31 are indicated by an arrow and shown at higher magnification in insert. Asterisks indicate base of ectopic HFs encircling dermal papilla (F, S, N, X). Scale bars: 100 µm (A–D), 50 µm (E–X).

To examine whether β-catenin and the VDR also cooperated to promote hair follicle differentiation *in vivo* in adult epidermis, we tested the effect of EB1089 on 4OHT treated K14ΔNβ-cateninER transgenic mice from two founder lines, D2 and D4. Activation of β-catenin stimulates proliferation and anagen entry in both high (D4) and low (D2) transgenic copy number mouse lines [5], [6]. Proliferation is stimulated to a greater extent in the D4 line, whereas ectopic HF morphogenesis is more advanced in the D2 line. The doses of EB1089 and 4OHT used did not induce epidermal changes in wild type mice (Figure 3A).

When D4 transgenic mice were treated with 4OHT for 14 days, existing HFs enlarged and entered anagen and K17, which is normally confined to the ORS (Figure 3A), was expressed in the IFE and SGs (Figure 3B). Small K17 positive outgrowths appeared in the interfollicular epidermis, representing rudimentary ectopic follicles (Figure 3B and insert). When mice were treated with 4OHT and EB1089, enlargement and anagen entry of existing follicles was delayed and SG morphology was preserved (Figure 3C). The K17 positive outgrowths in the IFE were longer and resembled HFs more closely than in mice treated with 4OHT alone (Figure 3C and insert). This conclusion was based on analysis of tail skin from 16 mice treated with 4OHT alone and 16 mice treated with 4OHT+EB1089. When treatment of D4 mice with 4OHT and EB1089 was extended to 21 days, most pre-existing follicles entered normal anagen, and the major site of ectopic follicle formation switched from IFE to SGs (Figure 3D). We quantified the number of ectopic follicles in the IFE of each of 50 tail epidermal units [5] (Figure 3C). There was no significant difference in the number of ectopic follicles induced by 4OHT alone or in combination with EB1089.

Many of the genes identified as transcriptional targets of β-catenin/VDR complexes encode hair follicle proteins (Figure 2; Table S1; Figure S1), leading us to predict that ectopic follicles induced in the presence of EB1089 would be more highly differentiated than those induced by β-catenin activation alone. In support of this prediction, several proteins that were undetectable in ectopic follicles induced by 4OHT alone were strongly induced by the combination of EB1089 and 4OHT.. These included K15 (Figure 3E, F), PADI3 (Figure 3G, H), S1003A (Figure 3I, J), K35 (Figure 3M, N), K71 (Figure 3O, P), K34 (Figure 3Q, R), and Gli1 (Figure 3S, T). In addition, combined EB1089 and 4OHT treatment resulted in an increased proportion of cells in ectopic follicles that expressed K31 (Figure 3K, L), Dlx3 (Figure 3U, V) and CCAAT displacement protein (CDP) (Figure 3W, X), than treatment with 4OHT alone. Cells expressing each of the induced proteins were correctly positioned in the ectopic follicles according to whether the proteins are markers of the HF outer root sheath, inner root sheath or cortex (Figure S1). In addition, dermal papilla formation, visualised as alkaline phosphatase positive mesenchymal cells encircled by a ‘cup’ of keratinocytes, was stimulated by treatment with EB1089 (Figure S3E, F; Figure 3F, L, N, X). The VDR was expressed at the base of wild type anagen follicles (Figure S1C) and in ectopic follicles induced by 4OHT alone or in combination with EB1089 (Figure S3A, B), expression being more widespread than nuclear β-catenin (Figure S3C, D).

The effects of EB1089 were not accompanied by significant changes in proliferation in either the D2 or the D4 line, whether visualised by Ki67 labelling of epidermal whole mounts (Figure S3G–I) or by flow cytometric determination of the proportion of epidermal cells in S, G2+M of the cell cycle (Figure S3J, K). Thus EB1089 differs from cyclopamine treatment, which has previously been shown to improve ectopic HF morphogenesis in the D4 transgenic line by inhibiting Hedgehog-induced proliferation [5] (Figure S3J, K). We conclude that EB1089 promotes β-catenin induced hair follicle differentiation without affecting proliferation, and that the effect is due to induction of a set of β-catenin/VDR target genes that characterise the HF lineages.

### VDR is essential for β-catenin induction of adult hair differentiation

It has previously been reported that in VDR−/− epidermis there is a gradual decrease in the size of the stem cell compartment and that this correlates with a failure of β-catenin to induce proliferation required for anagen entry [16]. Our data suggest the alternative hypothesis that the primary role of VDR/β-catenin interactions is to promote transcription of genes associated with differentiation of the hair follicle lineages. To investigate this, we examined the consequences of VDR ablation by crossing VDR null mice with the D2 line of K14ΔNβ-cateninER transgenics.

**Figure 4:**
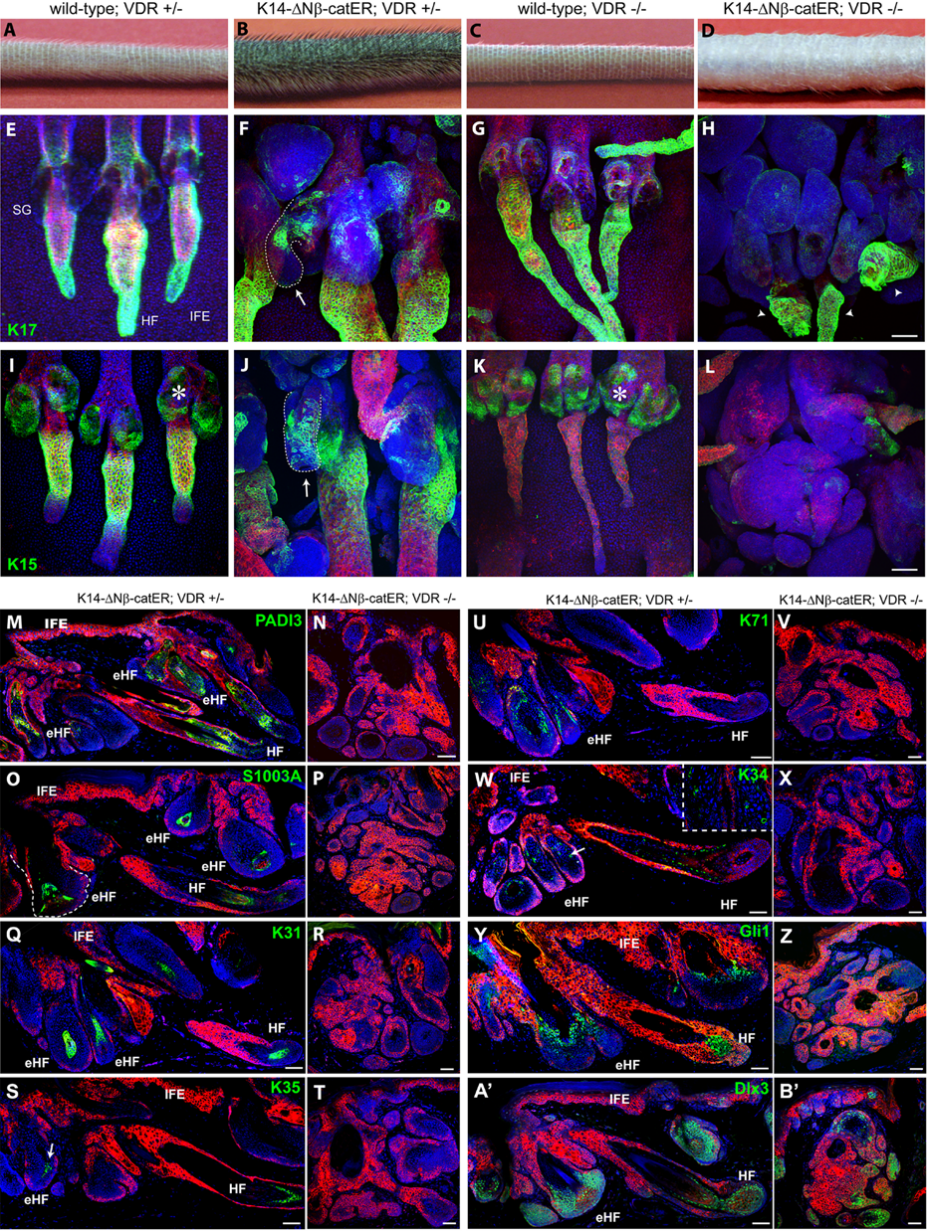
VDR is necessary for differentiation of β-catenin induced hair follicles. Mice were wild-type or D2 K14ΔNβ-cateninER transgenics, heterozygous (+/−) or homozygous (−/−) null for the VDR and had been treated with 4OHT for 21 days. (A–D) Macroscopic views of tail skin showing extent of hair growth. (E–L) Tail epidermal whole mounts immunolabelled with the antibodies indicated (green) and counterstained with Hoechst (blue) and phalloidin-TRICT (red). Arrows and dotted lines indicate ectopic HFs arising from SGs and arrowheads indicate residual HFs. Asterisks show nonspecific SG staining. (M-B') Immunostaining of tail skin sections with the antibodies indicated (green) and anti-keratin 14 (red) with Hoechst counterstain (blue). Scale bars: 100 µm.

VDR heterozygous mice were indistinguishable from wild type (Figure 4A). Although in some VDR−/− mice alopecia develops as early as 3 weeks after birth [20], alopecia was not yet evident in our 8 week old VDR −/− mice (Figure 4C and data not shown). Nevertheless, the HFs were thin and elongated. Although K17 was expressed (Figure 4G) K15 was undetectable (Figure 4K), providing confirmation of our finding that both β-catenin and VDR must be activated in order for the K15 gene to be transcribed (Figure 2). The absence of VDR was confirmed by antibody staining (Figure S4A, B).

K14ΔNβ-cateninER mice that were heterozygous for VDR were indistinguishable from D2 mice on a wild type background: 4OHT induced existing follicles to enter anagen, resulting in lengthening of the hairs on the tail (Figure 4B), and ectopic follicles formed, primarily from the SGs (Figure 4F). In keeping with the more extensive ectopic HF morphogenesis in D2 than D4 transgenics, ectopic follicles in D2 mice that were heterozygous for the VDR contained cells expressing many transcriptional target genes of β-catenin/VDR complexes that are HF lineage markers, including K15, PADI3, S1003A, K31, K35, K71, K34, Gli1, and Dlx3 (Figure 4J, M, O, Q, S, U, W, Y, A'). In contrast, D2 K14ΔNβ-cateninER mice that were null for VDR did not enter anagen. The HFs and SGs became thickened, but instead of forming ectopic follicles the enlarged cell masses lacked the expression of all the hair specific target genes analyzed, except for Dlx3 which was detectable in a small number of cells (Figure 4D, H, L, N, P, R, T, V, X, Z, B'). Further evidence that ectopic follicle formation was inhibited came from the reduction in alkaline phosphatase positive dermal papilla cells (Figure S4C, D).

The inhibition of β-catenin induced hair follicle differentiation did not correlate with a failure of VDR −/− epidermis to proliferate in response to β-catenin. The proportion of proliferative cells, detected by Ki67 labelling or flow cytometry, was higher in VDR null than wild type epidermis, and the increase in proliferation on β-catenin activation was very similar in both VDR+/− and VDR−/− backgrounds (Figure S4E–I). Induction of cyclin D1, a direct Lef1/β-catenin target gene, was not significantly altered in K14ΔNβ-cateninER;VDR−/− versus K14ΔNβ-cateninER;VDR+/− mice, suggesting that the absence of VDR did not influence the general activity of TCF/Lef dependent transcription (Figure S4J, K).

### Ligand activated VDR is essential to prevent β-catenin induced tumorigenesis

**Figure 5:**
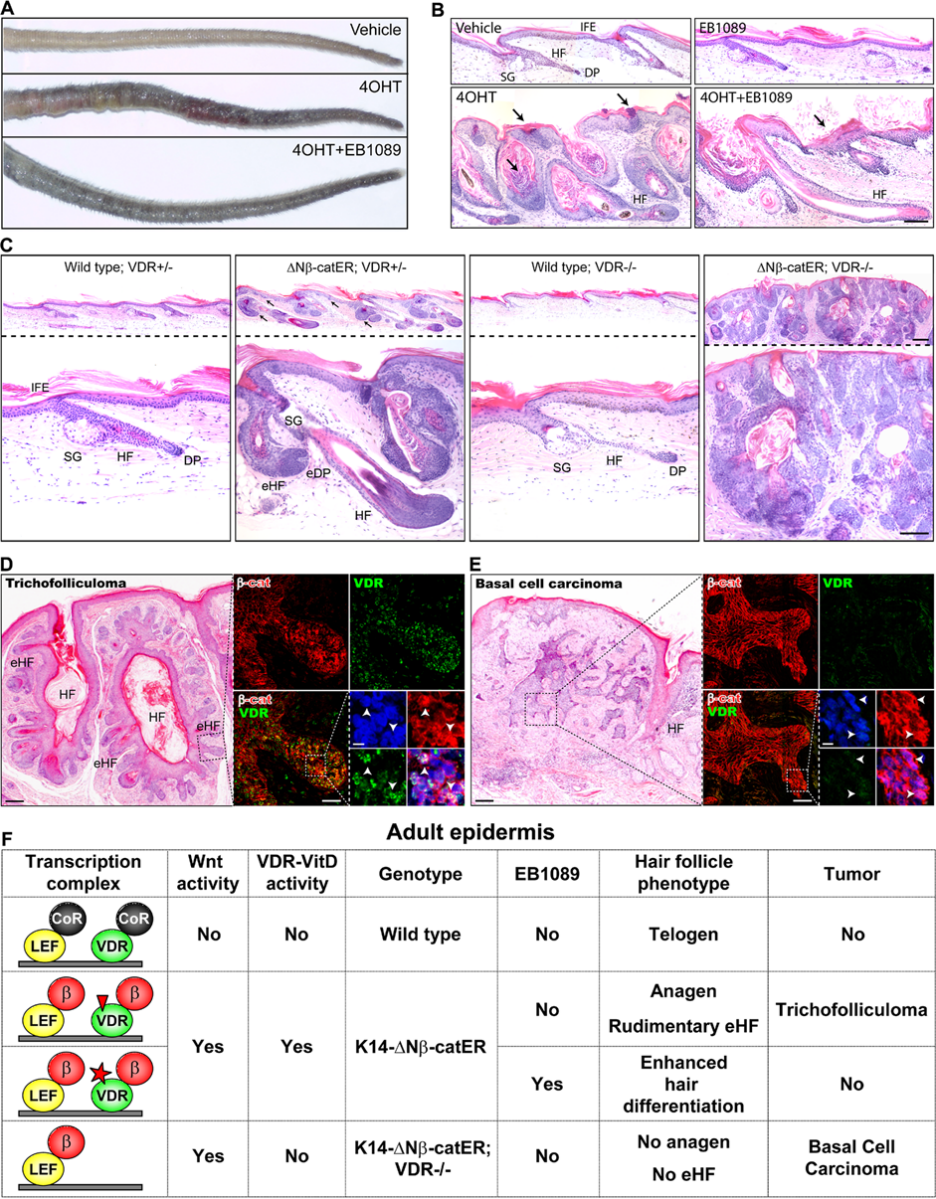
VDR modulates β-catenin induced skin tumours. (A, B) K14ΔNβ-cateninER (D4) transgenic mice were treated with vehicle, 4OHT, EB1089 or 4OHT+EB1089 for 21 days. (A) Macroscopic appearance of tails. (B) H&E stained tail skin sections. Arrows indicate parakeratosis and increased cornified layers. (C) H&E stained tail skin sections of wild type and K14ΔNβ-cateninER (D4) transgenic mice that were heterozygous (+/−) or homozygous (−/−) null for VDR, following 4OHT treatment for 21 days. Arrows indicate ectopic HF formation. eHF: ectopic hair follicles; DP: dermal papilla; eDP: ectopic dermal papilla. (D, E) Human trichofolliculoma (D) and infiltrative basal cell carcinoma (E). Serial sections were stained with H&E or immunolabelled for β-catenin (red) and VDR (green) with Hoescht counterstain (blue). Immunolabelling corresponds to boxed regions of H&E stained sections. Arrowheads show nuclear β-catenin and VDR. Scale bars: 100 µm (B–E), 50 µm (inserts in d, e), 10 µm (Hoescht staining inserts d,e). (F) Diagram summarizing dual role of β-catenin as a coactivator of TCF/Lef and VDR in adult epidermis. Hair follicle differentiation requires both the canonical Wnt pathway and ligand activated VDR. In the absence of VDR differentiation is impaired, favoring tumor formation. CoR: co-repressor; β: β-catenin; red triangle: endogenous vitamin D_3;_ red star: vitamin D analog EB1089. eHF: ectopic hair follicles.

If VDR/β-catenin interactions stimulate HF differentiation, then EB1089 may inhibit β-catenin induced tumour formation. Prolonged activation of β-catenin signalling in the D4 line of K14ΔNβ-cateninER transgenic mice results in the conversion of almost all of the follicles into benign tumours known as trichofolliculomas [6], and as a result the tails of 4OHT treated mice are swollen, lumpy and ulcerated (Figure 5A). Simultaneous treatment with EB1089 and 4OHT normalised the gross appearance of the tail (Figure 5A). Histological evaluation confirmed that EB1089 blocked trichofolliculoma development, inhibiting parakeratosis, stimulating IFE differentiation and restoring normal anagen (Figure 5B and data not shown). The protective effect of EB1809 was not due to inhibition of proliferation (Figure S3G–K). We conclude that EB1089 treatment inhibits the formation of HF tumours by promoting HF differentiated gene expression.

If the primary effect of VDR loss is to deplete the stem cell compartment [16] VDR −/− mice should be protected from β-catenin induced tumours. However, if VDR signalling promotes the HF lineages, then loss of VDR should change the type of tumour that forms in response to β-catenin [1]. To test these hypotheses we compared wild type;VDR+/−, K14ΔNβ-cateninER(D2);VDR+/−, wild type;VDR−/− and K14ΔNβ-cateninER(D2);VDR−/− mice, treated on the tail either with vehicle (acetone) or 4OHT for 21 days. All K14ΔNβ-cateninER;VDR−/− mice treated with 4OHT developed tumors (Figure 5C). The tumours did not have the histological appearance of trichofolliculomas and were negative for terminal differentiation markers of all three epidermal lineages: several hair keratins (HF), involucrin (IFE) and Oil Red (SG) (data not shown). We observed invasion of basal cells from the IFE and HF ORS, the cells having with large nuclei and small cytoplasm, which are features of BCC [21]. 18/18 4OHT treated K14ΔNβ-cateninER(D2);VDR−/− mice developed BCC-like tumors compared with 0/18 K14ΔNβ-cateninER(D2);VDR+/− mice, demonstrating a high penetrance of the phenotype.

To investigate whether or not nuclear β-catenin and VDR expression correlated with tumour type in human skin tumours, we examined a panel of 59 human tumours, which were categorised as basal cell carcinomas or having elements of HF differentiation (trichofolliculomas (TF), trichoepithelioma (TE), or sebaceous TF (STF) (Table S4). Those tumours with HF differentiation were characterised by high expression of both nuclear VDR and nuclear β-catenin, particularly within the aberrant follicles (Figure 5D). In contrast, all BCCs with low VDR expression and detectable nuclear β-catenin were characterized as infiltrative tumours (Figure 5E; Table S4). We conclude that low levels of VDR correlate with infiltrative BCCs (p<0.001).

## Discussion

Our results are summarised schematically in Figure 5F. In the absence of active Wnt or VDR signalling adult epidermal hair follicles are in the resting (telogen) phase of the hair growth cycle. In wild type skin the combined activation of Wnt and VDR by their endogenous ligands is required for normal anagen. Elevated and sustained activation of β-catenin in adult K14ΔNβ-cateninER epidermis leads to ectopic hair follicle formation and, subsequently, to trichofolliculomas [5], [6]. In the presence of EB1089 differentiation of ectopic HFs is stimulated and trichofolliculloma development is therefore blocked. Conversely, in the absence of VDR differentiation of ectopic follicles is inhibited and the tumours that develop in response to β−catenin are undifferentiated basal cell carcinomas.

It has recently been reported that the mechanism by which VDR loss leads to postnatal alopecia is via progressive stem cell depletion [16]. Stem cell depletion results in a failure of replacement of differentiated cells and, as a result, spontaneous wounds develop [22]. In contrast, as VDR −/− mice age they develop massively wrinkled skin, more consistent with expansion of the stem cell compartment (data not shown). Furthermore, we saw no evidence that VDR loss led to an impaired proliferative response to β-catenin, in contrast to the effects of inhibiting Hedgehog signalling [5]. All our data are consistent with the conclusion that combined activation of VDR and Wnt signalling promotes formation of TCF/Lef-β-catenin and VDR/β-catenin complexes that together induce genes that mediate HF differentiation.

β-catenin can no longer be considered as chiefly an activator of TCF/Lef target genes [23]. The interaction of β-catenin with other cofactors, such as VDR and Prop-1, is likely to contribute to the pleiotropic effects of the Wnt pathway, which has different target genes in different tissues. One example is the specification of the hair follicle lineages by β-catenin/VDR complexes. A second is the role of β-catenin binding to Prop-1 in cell lineage selection during mouse pituitary development [24]. In the skin, the ability of β-catenin to activate or inhibit other signalling pathways, such as Notch and Hedgehog, provides further levels of complexity in the regulation of stem cell renewal and lineage selection [25].

In human skin tumours with detectable nuclear β-catenin, the level of VDR correlates with differences in tumour phenotype, a similar situation to human colon cancer [26], [27]. These results suggest that alterations in VDR-β-catenin interactions, in combination with mutations in genes such as PTCH or p53, can modulate BCC development or progression. Vitamin D analogues may well be beneficial in the treatment of tumours in which the canonical Wnt pathway is activated inappropriately [28].

## Materials and Methods

### Antibodies, reagents and cell culture

The antibodies used have been described previously [5], except for VDR (Chemicon), β-catenin (BD Transduction), cytokeratins 14, 31, 34, 35, 71 (Progen), Dlx3, S1003A (Abnova), cyclin D1 (Abcam) and β-tubulin (Sigma). CDP antibody was a gift from Dr. Patrick Michl and PADI3 antibody a gift from Dr. Michel Simon. In some experiments cells or tissues were counterstained with phalloidin (Sigma) and Hoechst 33258 (Molecular Probes).

Primary keratinocytes derived from wild type, VDR null and K14ΔNLef1 mice [29] were isolated and cultured as previously described [5]. Wnt 3A protein was purified as previously described [30]. EB1089 was a gift from Leo Pharmaceuticals.

### Bioinformatics and statistics

TCF/Lef and VDR variant consensus motifs were defined by comparing the natural binding sites in the promoter region of several target genes (Table S2). The sites were mapped to 3 kb of promoter sequence of the β-catenin target genes using the Emboss program fuzznuc [31] allowing for 1 mismatch. Identified motifs were filtered on conservation between mouse and human [32]. To determine over-representation of motifs within the gene list, a background was constructed by mapping consensus motifs to 3 kb of promoter sequence for all NCBI reference sequences (RefSeq). This sequence set was then randomly sampled to derive a background distribution against which the β-catenin target gene motif numbers were tested (p-values).

In evaluating human tumour sections we calculated the probability of infiltrative tumours having low levels of VDR and detectable levels of nuclear β-catenin and performed a contingency analysis using the ChiSquare Test (p-values).

### Generation and experimental treatment of mice

The D2 and D4 lines of K14ΔNβ-cateninER transgenic mice have been described previously [5], [6]. VDR −/− mice were fed with a special diet to prevent rickets (Yoshizawa [20]. At the start of each experiment, mice were 6 to 8 weeks old, and therefore in the resting phase of the hair cycle. VDR−/− mice started to develop alopecia at 9 weeks of age, which later than some other VDR null strains [15], [20], [33].

The K14ΔNβ-cateninER transgene was activated by topical application of 4-hydroxytamoxifen (4OHT; Sigma) dissolved in acetone. Tail skin was treated by applying 4OHT with a paint brush (0.5 mg per mouse every second day). In some experiments, mice received topical applications of EB1089 (1.5 mg in acetone per mouse) 30 min prior 4OHT treatment. All animal experiments had the approval of the CR-UK London Research Institute ethics committee and were carried out under a British Home Office license.

Three independent experiments comparing D4 line of K14ΔNβ-cateninER transgenics and transgenic negative littermates (wild type) were performed. The combined total of mice per treatment group and genotype was 16.

Four independent experiments examining the D2 line of K14ΔNβ-cateninER of transgenic mice on a VDR−/+ or VDR−/− backgrounds, were performed. A total of 18 mice per treatment group/genotype was examined.

### Immunoprecipitation, immunoblotting and luciferase assays

Primary mouse keratinocytes were grown in KSFM medium (Invitrogen-Gibco) and deprived of serum overnight prior to treatment. Cell lysis, immunoprecipitation and immunoblotting were performed as described [10], except that immunoprecipitates were washed five times with 150 mM NaCl, 10% glycerol, 1% Triton-X100 and 50 mM Hepes, pH 7.4. For luciferase assays, keratinocytes were transiently transfected with Promega luciferase reporter construct pRL (Renilla luciferase control) and TOPFLASH, FOPFLASH, 4×VDRE or −5 kb mouse Krt15 promoter (firefly luciferase) using FuGene 6 (Roche) as previously described [10] and treated with 10^−7 ^M EB1089 and/or 100 ng/ml Wnt3A. In some experiments cells were cotransfected with pSG5-VDR or pSG5 (Mock) constructs. Luciferase activity was measured using the PerkinElmer EnVision™ system.

### RNA extraction and real time PCR

Total RNA from primary mouse keratinocytes was purified using the Tri Reagent – RNA/DNA/Protein Isolation Procedure (Helena BioSciences). Retrotranscription of mRNA to cDNA was performed using 0.5 µg of total RNA from each sample, 2.5 µM oligo dT primer (5′tttttttttttttt 3′), 0.5 mM dNTP mix, 0.05 mM DTT, 4 µl of 5× First Strand Buffer (Invitrogene) and 1 µl of Retrotranscriptase (Invitrogene) in a final 20 µl reaction. All samples where analyzed by real-time PCR using SYBR Green PCR master mix (Applied Biosystems) and specific pairs of primers for each gene (Table S5).

### Chromatin immunoprecipitation assay

Cells were treated with 10^−7^ M EB1089 and/or 100 ng/ml Wnt3A for 4 h. 10^6^ keratinocytes were lysed and immunoprecipitated with 2.5 µg of VDR (Sigma), β-catenin (BD Transduction), Lef1 (Upstate Biotech) or unrelated HA (Roche) antibodies. Sonication, precipitation and washes were performed using an Upstate ChIP assay kit. All the samples where analysed by real-time PCR using SYBR Green PCR master mix (Applied Biosystems) and specific pairs of primers for two regions of each promoter that contained VDRE and/or TCF/Lef binding sites, one region that does not contain any of these sites, and a completely unrelated region of the genome as a negative control (Table S3). The sequences of the binding sites for each promoter are listed in Table S4. PCR cycles were as follows: 94°C for 5 min; 40 cycles of (95°C 15 sec, 60°C 20 sec, 72°C 15 sec), 72°C for 5 min, and a final 19.59 min of ramping the temperature to 95°C in order to calculate the dissociation curve of the PCR products. Real-Time PCR was performed with the ABI 7700 Real-Time PCR system (Applied Biosystems). The primers used are indicated in Table S5. All 64 samples were run in the same 96 well plate to allow us to compare the different levels of amplification.

Real-time PCR was repeated more than three times within every experiment and each experiment was repeated three times. To calculate the fold values we first measured the cycle number at which the increase in fluorescence (and therefore cDNA) was logarithmic. The point at which the fluorescence crossed the threshold is called the Ct. We corrected the Ct values of each sample by the Ct value obtained for the corresponding input using the same primers or using unrelated primers. The formula used is shown as follows:

1. a : Ct value for PCR of immunoprecipitated samples.
2. i : Ct value for PCR of imput samples (prior immunoprecipitation).
3. c : Ct value for PCR using unrelated oligos for each sample.
4. DctIP : Differential Ct for the immunoprecipitates. DctIP = a-i
5. DctC : Differential Ct control. DctC = c-i
6. DDct : Differential between DctIP and DctC. DDct = DctIP-DctC.
7. Fold = 2^DDct^

### Immunohistochemistry

Conventional fixed, paraffin-embedded sections and tail epidermal whole mounts were prepared and immunolabeled as described previously [5]. Images were obtained using a Zeiss 510 confocal microscope [34]. Alkaline phosphatase activity was measured on frozen sections as previously described [6]. Human tumor samples were obtained with ethical approval from Yamagata University Hospital.

### Flow cytometry

To analyze cell cycle keratinocytes isolated from epidermis were fixed with 2% paraformaldehyde (PFA) at room temperature for 10 min and then permeabilized with 0.1% Triton-X100 for 10 min. Cells were washed with PBS and re-suspended in PBS with 2% FBS containing DAPI (2 mg/ml; Sigma). Pulse processing was used in order to exclude any unstained, apoptotic or clumped cells and analysis was performed using an LSRII (Becton-Dickinson). Analysis of flow cytometry data was performed using a FlowJo 6.3.3 (Treestar Inc., Ashland, Oregon).

## Supporting Information

Figure S1 Expression of beta-catenin target genes in wild type anagen follicles. (A) H&E staining. Positions of dermal papilla (DP), hair matrix (ma), outer root sheath (ORS), inner root sheath (IRS) and hair shaft (HS) are indicated. (B-L) Immunostaining with antibodies indicated (green), anti-keratin 14 (red) and Hoescht (blue) counterstain. Scale bar: 100 micrometers.
(9.52 MB TIF)

Figure S2 Beta-catenin is recruited to hair follicle gene promoters by binding ligand activated VDR. (A,B) Wild type (WT; white bars) and VDR null (KO; black bars) primary mouse keratinocytes were lysed and immunoprecipitated with VDR, beta-catenin or unrelated antibody (HA tag; UR). Immunoprecipitated genomic DNA fragments or input controls were amplified by real-time PCR using specific primers for region 3 of the mouse promoters indicated or unrelated genomic primers (UR). Data are means±S.D. of triplicate reactions. Scaled diagrams summarize location of VDREs and TCF/Lef binding sites and the proteins bound to each region in cells treated with Wnt3A and EB1089.
(0.91 MB TIF)

Figure S3 EB1089 promotes ectopic hair follicle differentiation without affecting proliferation in K14DeltaNbeta-cateninER transgenic mice. Epidermal sections (A–F) or whole mounts (G–H) of D4 tail skin treated with 4OHT and/or EB1089. (A–D) Double immunolabelling with keratin 14 (red) and the antibodies shown (green), with Hoechst (blue) counterstain. Asterisks indicate ectopic HFs encircling dermal papillae. Dashed lines demarcate dermal-epidermal boundary. (E, F) Alkaline phosphatase activity (blue) with fast red counterstain. Asterisk indicates dermal papilla. (G–I) Ki67 staining (green) with phalloidin-TRITC (red) counterstain. Inserts show IFE sections. Scale bars: 50 micrometers (A–F). (J, K) DNA content of keratinocytes isolated from mouse skin was used to determine proportion of cells in different phases of the cell cycle. % cells in S+G2/M phase was calculated for mice treated as indicated. Cyclop: cyclopamine. Data shown are for two mice of each founder line per treatment.
(5.85 MB TIF)

Figure S4 Lack of VDR impairs beta-catenin induced hair follicle differentiation but not proliferation. D2 mice were treated with 4OHT for 21 days and tail epidermis was analyzed. (A, B) Double immunostaining of tail skin sections with antibodies to keratin 14 (red) and VDR (green) with Hoechst counterstain (blue). (C, D) Alkaline phosphatase activity (blue) with Fast Red counterstain. Dashed line in (C) indicates dermal-epidermal junction. (E–H) Whole mount staining for Ki67 with Hoescht (blue) and phalloidin (red) counterstains. (I) % cells in S+G2/M was determined by flow cytometry. Data shown are for two mice of each genotype. (J, K) Immunohistochemistry for cyclin D1 (brown). Positive staining is indicated by arrows. eHF: ectopic hair follicle; eDP: ectopic dermal papilla. Scale bars: 100 micrometers.
(5.18 MB TIF)

Table S1 TCF/Lef and VDR binding sites in the promoter regions of beta-catenin target genes. The 3 kb proximal promoter region of 91 genes upregulated more than 3 fold in transgenic skin of K14DeltaNbeta-cateninER (D2) mice treated with 4OHT for 7 days [5] was analyzed. The numbers of putative TCF/Lef and VDR variant consensus motifs, filtered on conservation between mouse and human, are shown [19]. The list is organized into different groups according to the abundance of VDREs and TCF/Lef binding sites. Within each group genes are ranked according to fold upregulation on the original microarrays. Genes with multiple LEF and VDR sites are subdivided according to whether they have fewer TCF/Lef sites than VDREs, similar numbers of both types of sites or lower number of VDREs than TCF/Lef sites.
(0.03 MB DOC)

Table S2 Enrichment of TCF/Lef and VDR binding sites in the promoter of beta-catenin target genes. To determine over-representation of motifs within the gene list in Table S1, a background was constructed by mapping consensus motifs to 3 kb of promoter sequence for all NCBI reference sequences (RefSeq). This sequence set was then randomly sampled to derive a background distribution against which the beta-catenin target gene motif numbers were tested (p-values). The total number of TCF/Lef binding sites (303) was calculated for the 91 genes studied (Table S1) [19]. The presence of 11 different VDR binding motifs was analyzed in the same 91 genes. 5 types of VDREs were significantly enriched in the gene list (p<0.05), with 414 sites present. The presence of the other VDREs (55 sites) was not significantly increased in the gene list (p>0.05). The references listed correspond to the original reports of natural TCF/Lef and VDR binding sites, used to define the consensus motifs. The consensus motifs use a degenerate code: A = A, C = C, G = G, T = T, R = AG, Y = CT, M = AC, K = GT, W = AT, S = CG, B = CGT, D = AGT, H = ACT, V = ACG, N = ACGT.
(0.04 MB DOC)

Table S3 VDR and Lef1 binding sites in the promoter regions of Krt15, PADI3 and S1003A genes. For each gene the actual sequence present in the mouse promoter is shown (real site), together with the corresponding consensus binding site and the regions in the ChIP analysis. Sequences are numbered in order, according to their relative proximity to the transcription start (number 1 being closest). DR: Direct Repeat. IP: inverted palindrome.
(0.07 MB DOC)

Table S4 VDR and nuclear beta-catenin expression in human skin tumours. 59 human skin tumors were stained for VDR and beta-catenin. Expression was scored as high, medium or low. In green are highlighted all tumors with elements of hair follicle differentiation; these had high levels of both VDR and nuclear beta-catenin. In red are indicated those infiltrative BCCs that had high nuclear beta-catenin and low VDR.
(0.08 MB DOC)

Table S5 Primers used for real time PCR
(0.02 MB DOC)
