# Supplementary figures and images for: The Vitamin D Receptor Is a Wnt Effector that Controls Hair Follicle Differentiation and Specifies Tumor Type in Adult Epidermis
Source: PLoS One. 2008 Jan 23;3(1):e1483. doi: 10.1371/journal.pone.0001483 (PMC2198947; doi:10.1371/journal.pone.0001483)

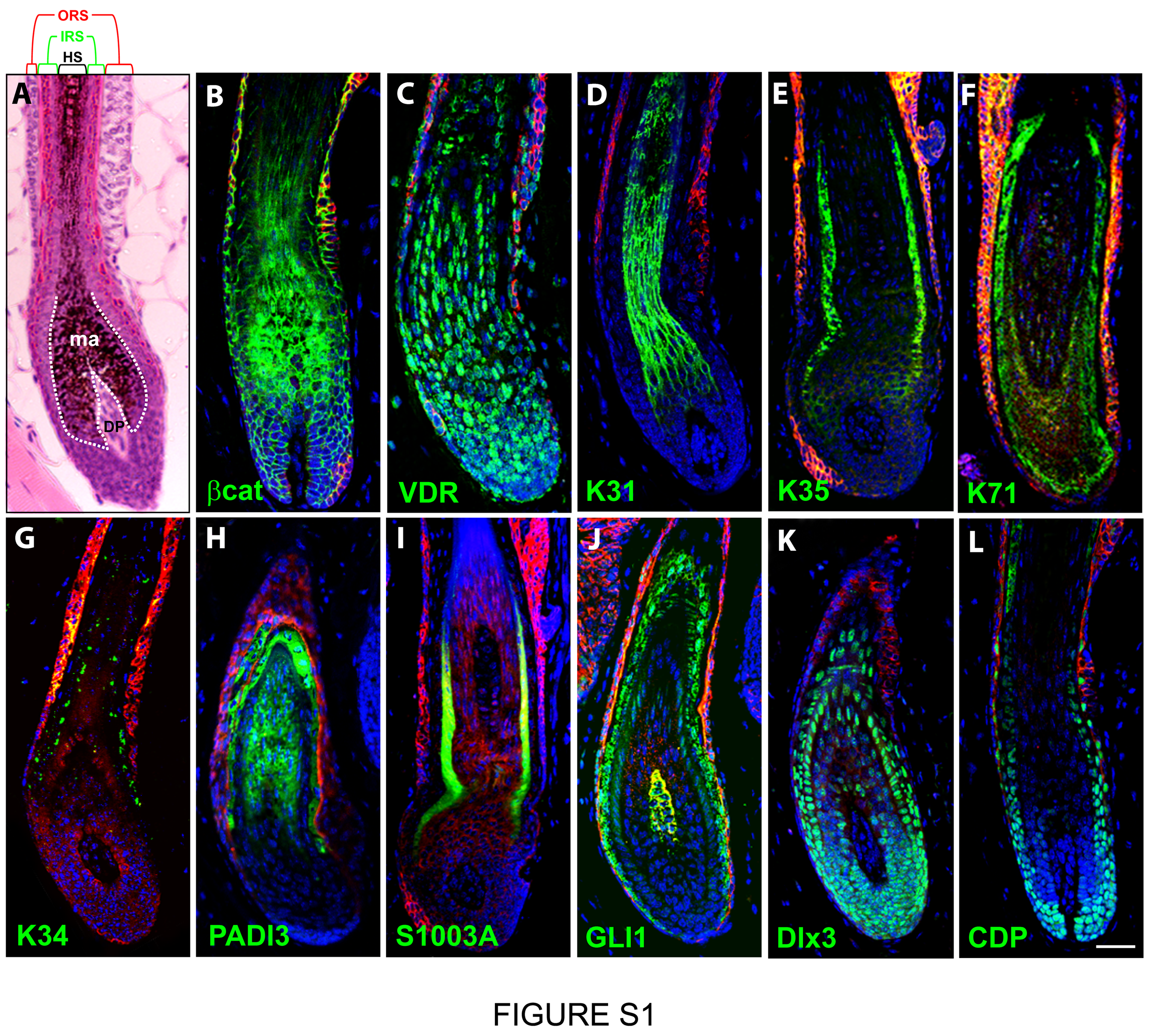

Supplement: Figure S1 — Expression of beta-catenin target genes in wild type anagen follicles. (A) H&E staining. Positions of dermal papilla (DP), hair matrix (ma), outer root sheath (ORS), inner root sheath (IRS) and hair shaft (HS) are indicated. (B-L) Immunostaining with antibodies indicated (green), anti-keratin 14 (red) and Hoescht (blue) counterstain. Scale bar: 100 micrometers. (9.52 MB TIF) [file pone.0001483.s001.tif]

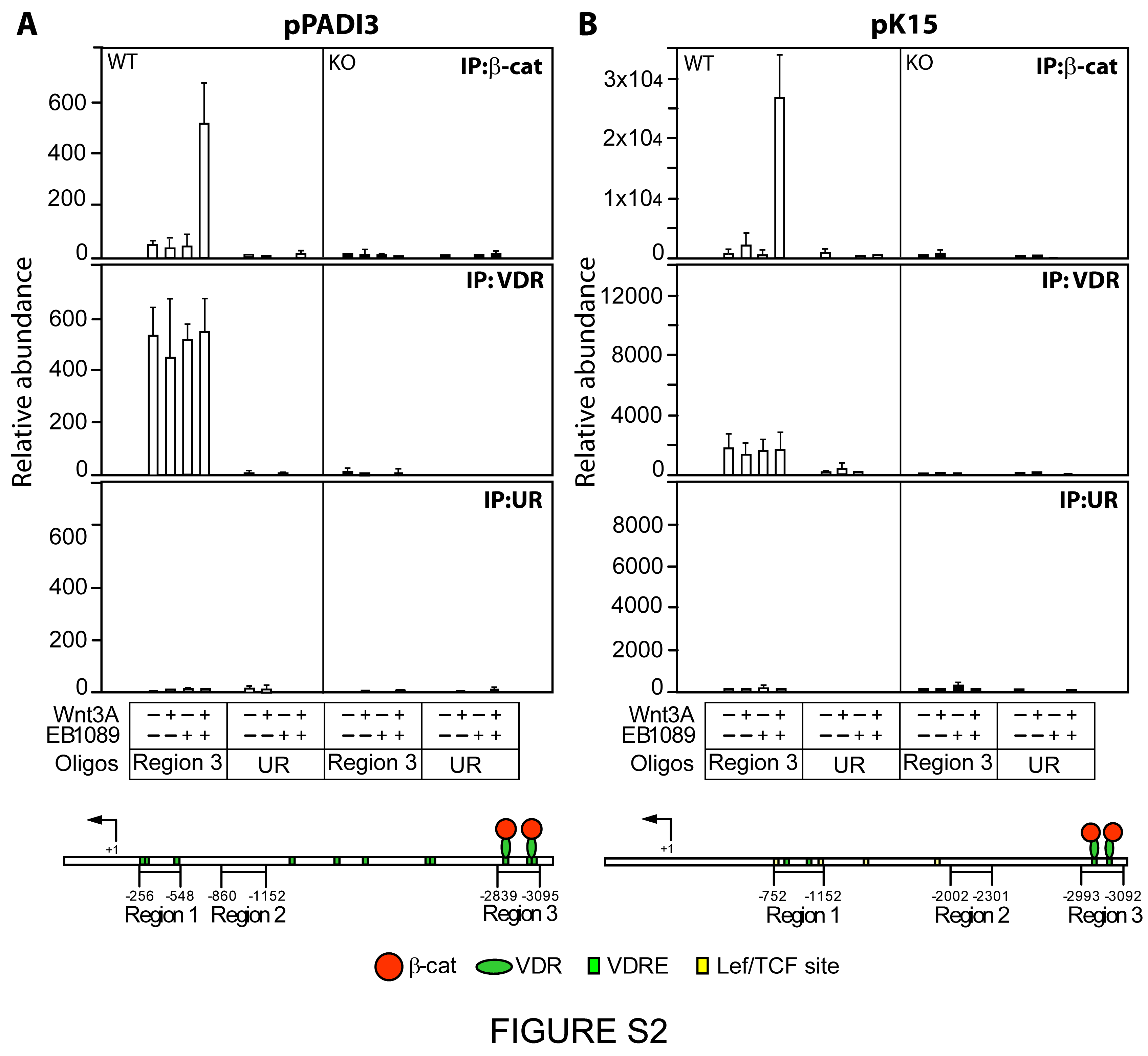

Supplement: Figure S2 — Beta-catenin is recruited to hair follicle gene promoters by binding ligand activated VDR. (A,B) Wild type (WT; white bars) and VDR null (KO; black bars) primary mouse keratinocytes were lysed and immunoprecipitated with VDR, beta-catenin or unrelated antibody (HA tag; UR). Immunoprecipitated genomic DNA fragments or input controls were amplified by real-time PCR using specific primers for region 3 of the mouse promoters indicated or unrelated genomic primers (UR). Data are means±S.D. of triplicate reactions. Scaled diagrams summarize location of VDREs and TCF/Lef binding sites and the proteins bound to each region in cells treated with Wnt3A and EB1089. (0.91 MB TIF) [file pone.0001483.s002.tif]

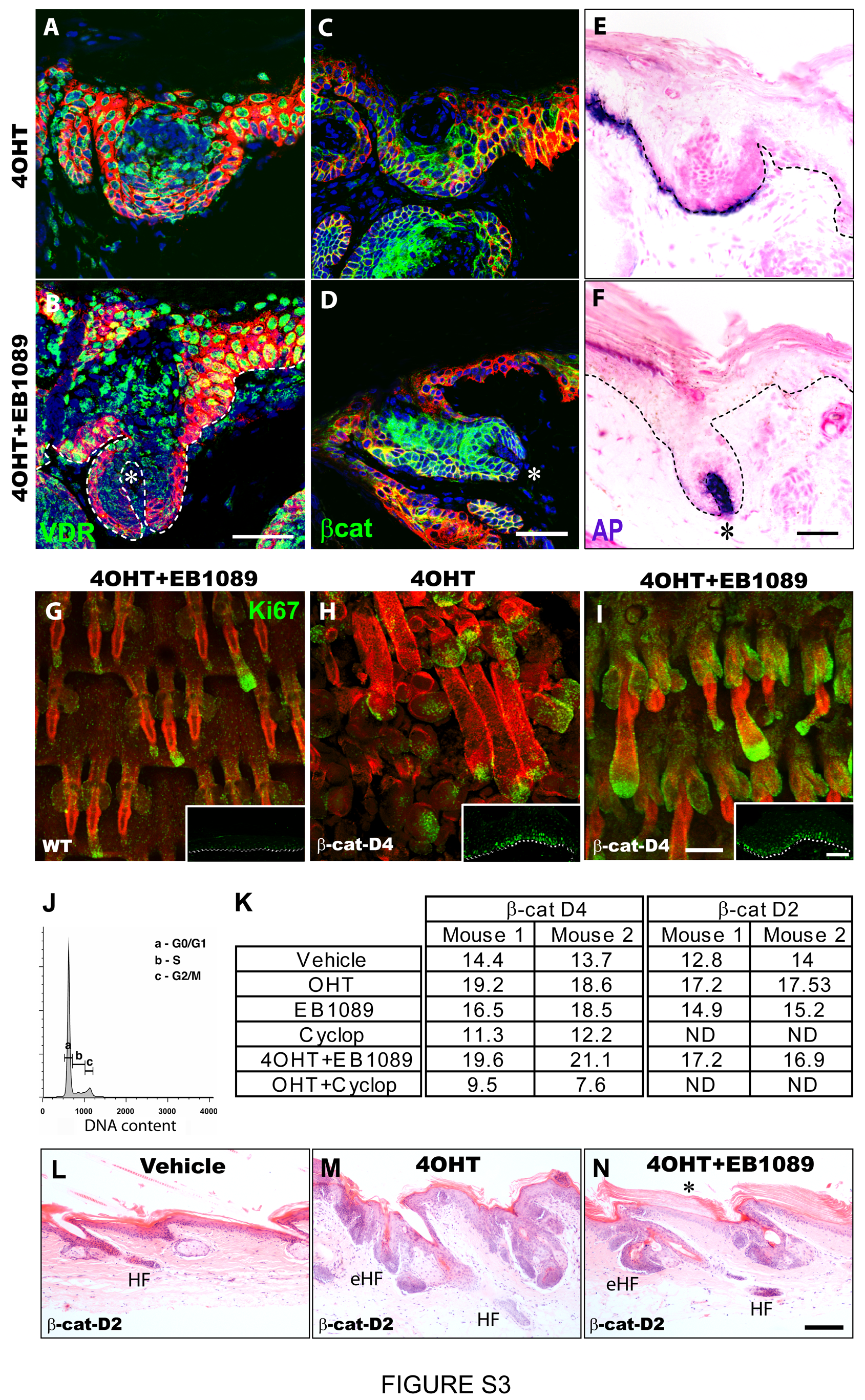

Supplement: Figure S3 — EB1089 promotes ectopic hair follicle differentiation without affecting proliferation in K14DeltaNbeta-cateninER transgenic mice. Epidermal sections (A–F) or whole mounts (G–H) of D4 tail skin treated with 4OHT and/or EB1089. (A–D) Double immunolabelling with keratin 14 (red) and the antibodies shown (green), with Hoechst (blue) counterstain. Asterisks indicate ectopic HFs encircling dermal papillae. Dashed lines demarcate dermal-epidermal boundary. (E, F) Alkaline phosphatase activity (blue) with fast red counterstain. Asterisk indicates dermal papilla. (G–I) Ki67 staining (green) with phalloidin-TRITC (red) counterstain. Inserts show IFE sections. Scale bars: 50 micrometers (A–F). (J, K) DNA content of keratinocytes isolated from mouse skin was used to determine proportion of cells in different phases of the cell cycle. % cells in S+G2/M phase was calculated for mice treated as indicated. Cyclop: cyclopamine. Data shown are for two mice of each founder line per treatment. (5.85 MB TIF) [file pone.0001483.s003.tif]

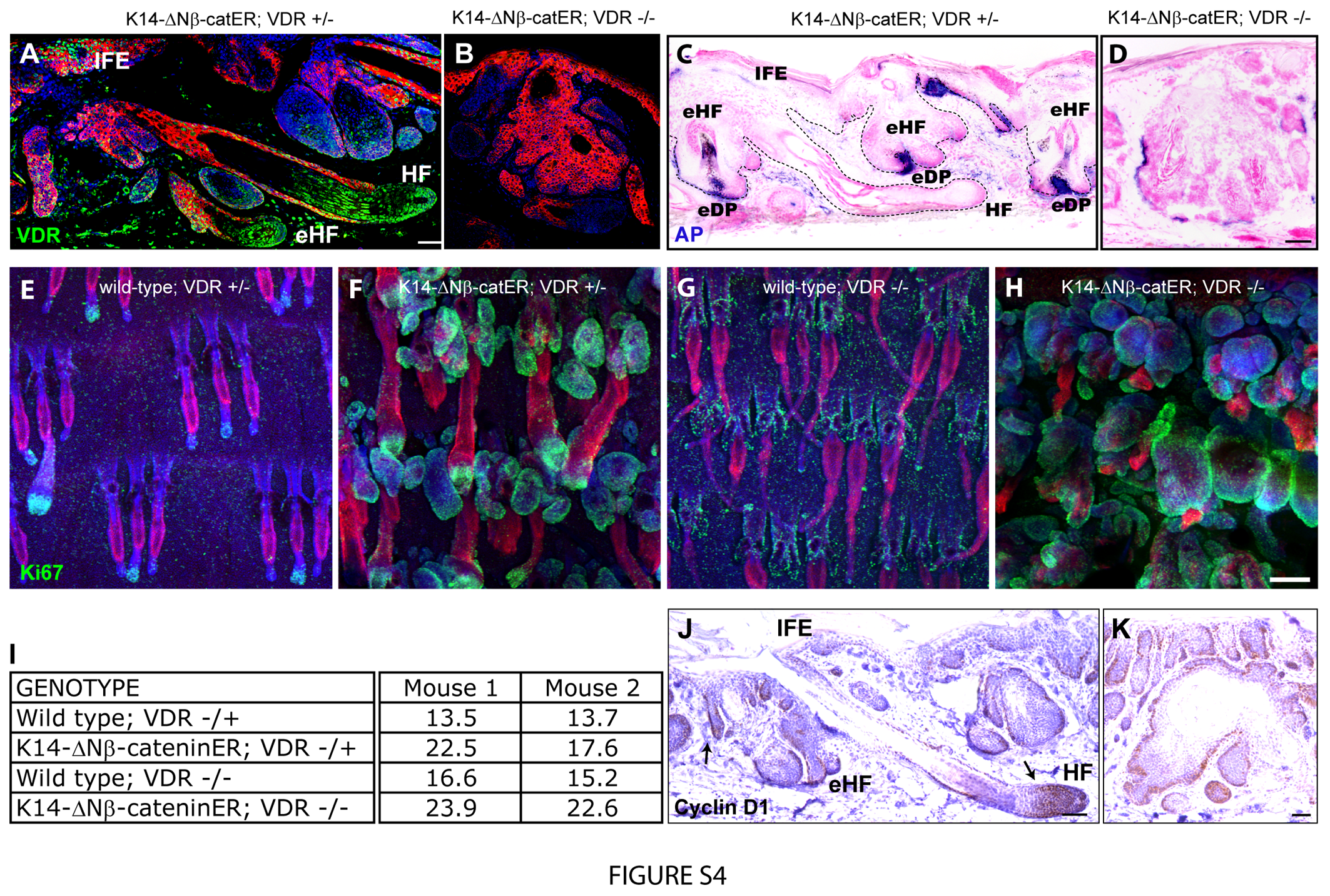

Supplement: Figure S4 — Lack of VDR impairs beta-catenin induced hair follicle differentiation but not proliferation. D2 mice were treated with 4OHT for 21 days and tail epidermis was analyzed. (A, B) Double immunostaining of tail skin sections with antibodies to keratin 14 (red) and VDR (green) with Hoechst counterstain (blue). (C, D) Alkaline phosphatase activity (blue) with Fast Red counterstain. Dashed line in (C) indicates dermal-epidermal junction. (E–H) Whole mount staining for Ki67 with Hoescht (blue) and phalloidin (red) counterstains. (I) % cells in S+G2/M was determined by flow cytometry. Data shown are for two mice of each genotype. (J, K) Immunohistochemistry for cyclin D1 (brown). Positive staining is indicated by arrows. eHF: ectopic hair follicle; eDP: ectopic dermal papilla. Scale bars: 100 micrometers. (5.18 MB TIF) [file pone.0001483.s004.tif]
