# Supplementary material for: The Vitamin D Receptor Is a Wnt Effector that Controls Hair Follicle Differentiation and Specifies Tumor Type in Adult Epidermis
Source: PLoS One. 2008 Jan 23;3(1):e1483. doi: 10.1371/journal.pone.0001483 (PMC2198947; doi:10.1371/journal.pone.0001483)
Supplement: Table S1 — TCF/Lef and VDR binding sites in the promoter regions of beta-catenin target genes. The 3 kb proximal promoter region of 91 genes upregulated more than 3 fold in transgenic skin of K14DeltaNbeta-cateninER (D2) mice treated with 4OHT for 7 days [5] was analyzed. The numbers of putative TCF/Lef and VDR variant consensus motifs, filtered on conservation between mouse and human, are shown [19]. The list is organized into different groups according to the abundance of VDREs and TCF/Lef binding sites. Within each group genes are ranked according to fold upregulation on the original microarrays. Genes with multiple LEF and VDR sites are subdivided according to whether they have fewer TCF/Lef sites than VDREs, similar numbers of both types of sites or lower number of VDREs than TCF/Lef sites. (0.03 MB DOC) [file pone.0001483.s005.doc]

**TABLE S1**
