# Supplementary material for: The Vitamin D Receptor Is a Wnt Effector that Controls Hair Follicle Differentiation and Specifies Tumor Type in Adult Epidermis
Source: PLoS One. 2008 Jan 23;3(1):e1483. doi: 10.1371/journal.pone.0001483 (PMC2198947; doi:10.1371/journal.pone.0001483)
Supplement: Table S2 — Enrichment of TCF/Lef and VDR binding sites in the promoter of beta-catenin target genes. To determine over-representation of motifs within the gene list in Table S1, a background was constructed by mapping consensus motifs to 3 kb of promoter sequence for all NCBI reference sequences (RefSeq). This sequence set was then randomly sampled to derive a background distribution against which the beta-catenin target gene motif numbers were tested (p-values). The total number of TCF/Lef binding sites (303) was calculated for the 91 genes studied (Table S1) [19]. The presence of 11 different VDR binding motifs was analyzed in the same 91 genes. 5 types of VDREs were significantly enriched in the gene list (p<0.05), with 414 sites present. The presence of the other VDREs (55 sites) was not significantly increased in the gene list (p>0.05). The references listed correspond to the original reports of natural TCF/Lef and VDR binding sites, used to define the consensus motifs. The consensus motifs use a degenerate code: A = A, C = C, G = G, T = T, R = AG, Y = CT, M = AC, K = GT, W = AT, S = CG, B = CGT, D = AGT, H = ACT, V = ACG, N = ACGT. (0.04 MB DOC) [file pone.0001483.s006.doc]

| **TABLE S2** | |  |  |
| --- | --- | --- | --- |
|  |  |  |  |
| Transc.Factor | Motif | P-value | Total motifs |
| LEF1 | CTTTGWW | 0 | 303 |
|  |  |  |  |
| VDR | TSVMMYNNNNNNNNNRKKBSA | 6,66E-016 | 57 |
| p<0.05 | RKKBSANNNTSVMMY | 1,41E-007 | 72 |
|  | RKKBSANNNNNNRKKBSA | 0,000606483 | 88 |
|  | TSVMMYNNNNNNNRKKBSA | 0,002710991 | 62 |
|  | TSVMMYNNNNNNNNNNNRKKBSA | 0,021437809 | 52 |
|  | RKKBSANNNRKKBSA | 0,042078137 | 83 |
|  |  |  | 414 |
|  |  |  |  |
| VDR | RGKTSANNNNNNRGKTSA | 0,836596487 | 5 |
| p>0.05 | RGKTSANNNNRGKTSA | 0,616461392 | 4 |
|  | RGKTSANNNRGKTSA | 0,849306522 | 1 |
|  | RGKTSANNNTSAMCY | 0,19891525 | 3 |
|  | TSAMCYNNNNNNNNNNNNRGKSTA | 0,432177599 | 1 |
|  | TSAMCYNNNNNNNNNNNRGKSTA | 0,142170591 | 2 |
|  | TSAMCYNNNNNNNRGKSTA | 0,45756116 | 1 |
|  | TSVMMYNNNNNNNNNNNNRKKBSA | 0,539232628 | 38 |
|  |  |  | 55 |

**LEF1 consensus motifs**

[Giese, K., Amsterdam, A. & Grosschedl, R.](http://www.ncbi.nlm.nih.gov/entrez/query.fcgi?db=pubmed&cmd=Retrieve&dopt=AbstractPlus&list_uids=1752444&query_hl=19&itool=pubmed_docsum) DNA-binding properties of the HMG domain of the lymphoid-specific transcriptional regulator LEF-1. *Genes Dev* **5**, 2567-78 (1991).

[Hardman, C.H. et al.](http://www.ncbi.nlm.nih.gov/entrez/query.fcgi?db=pubmed&cmd=Retrieve&dopt=AbstractPlus&list_uids=8527432&query_hl=24&itool=pubmed_docsum) Structure of the A-domain of HMG1 and its interaction with DNA as studied by heteronuclear three- and four-dimensional NMR spectroscopy. *Biochemistry* **26**, 16596-607 (1995).

[van de Wetering M.](http://www.ncbi.nlm.nih.gov/entrez/query.fcgi?db=pubmed&cmd=Retrieve&dopt=AbstractPlus&list_uids=9118222&query_hl=26&itool=pubmed_docsum) et al. Armadillo coactivates transcription driven by the product of the Drosophila segment polarity gene dTCF.
*Cell* **21**, 789-99 (1997).

[van Beest, M.et al.](http://www.ncbi.nlm.nih.gov/entrez/query.fcgi?db=pubmed&cmd=Retrieve&dopt=AbstractPlus&list_uids=10867006&query_hl=28&itool=pubmed_docsum) Sequence-specific high mobility group box factors recognize 10-12-base pair minor groove motifs. *J Biol Chem* **275**, 27266-73 (2000).

**VDR consensus motifs**

[Turunen, M.M., Dunlop, T.W., Carlberg, C. & Vaisanen, S.](http://www.ncbi.nlm.nih.gov/entrez/query.fcgi?db=pubmed&cmd=Retrieve&dopt=AbstractPlus&list_uids=17426122&query_hl=12&itool=pubmed_docsum) Selective use of multiple vitamin D response elements underlies the 1 {alpha} ,25-dihydroxyvitamin D3-mediated negative regulation of the human CYP27B1 gene. *Nucleic Acids Res* **10** (2007).

[Saramaki A, Banwell CM, Campbell MJ, Carlberg C.](http://www.ncbi.nlm.nih.gov/entrez/query.fcgi?db=pubmed&cmd=Retrieve&dopt=AbstractPlus&list_uids=16434701&query_hl=4&itool=pubmed_docsum) Regulation of the human p21(waf1/cip1) gene promoter via multiple binding sites for p53 and the vitamin D3 receptor. *Nucleic Acids Res* **24**, 543-54 (2006).

[Matilainen, M., Malinen, M., Saavalainen, K. & Carlberg, C.](http://www.ncbi.nlm.nih.gov/entrez/query.fcgi?db=pubmed&cmd=Retrieve&dopt=AbstractPlus&list_uids=16186133&query_hl=4&itool=pubmed_docsum) Regulation of multiple insulin-like growth factor binding protein genes by 1alpha,25-dihydroxyvitamin D3. *Nucleic Acids Res* **26**, 5521-32. (2005).

[Vaisanen, S., Dunlop, T.W., Sinkkonen, L., Frank, C. & Carlberg, C.](http://www.ncbi.nlm.nih.gov/entrez/query.fcgi?db=pubmed&cmd=Retrieve&dopt=AbstractPlus&list_uids=15919092&query_hl=4&itool=pubmed_docsum) Spatio-temporal activation of chromatin on the human CYP24 gene promoter in the presence of 1alpha,25-Dihydroxyvitamin D3. *J Mol Biol* **1**, 65-77 (2005).

[Dunlop, T.W.](http://www.ncbi.nlm.nih.gov/entrez/query.fcgi?db=pubmed&cmd=Retrieve&dopt=AbstractPlus&list_uids=15890193&query_hl=4&itool=pubmed_docsum) et al. The human peroxisome proliferator-activated receptor delta gene is a primary target of 1alpha,25-dihydroxyvitamin D3 and its nuclear receptor. *J Mol Biol* **349**, 248-60 (2005).

[Sinkkonen, L., Malinen, M., Saavalainen, K., Vaisanen, S. & Carlberg, C.](http://www.ncbi.nlm.nih.gov/entrez/query.fcgi?db=pubmed&cmd=Retrieve&dopt=AbstractPlus&list_uids=15863722&query_hl=4&itool=pubmed_docsum) Regulation of the human cyclin C gene via multiple vitamin D3-responsive regions in its promoter. *Nucleic Acids Res* **33**, 2440-51 (2005).

[Dunlop, T.W., Vaisanen, S., Frank, C. & Carlberg, C.](http://www.ncbi.nlm.nih.gov/entrez/query.fcgi?db=pubmed&cmd=Retrieve&dopt=AbstractPlus&list_uids=15225781&query_hl=4&itool=pubmed_DocSum) The genes of the coactivator TIF2 and the corepressor SMRT are primary 1alpha,25(OH)2D3 targets*. J Steroid Biochem Mol Biol* **89-90**, 257-60. (2004).

[Schrader, M., Nayeri, S., Kahlen, J.P., Muller, K.M. & Carlberg, C.](http://www.ncbi.nlm.nih.gov/entrez/query.fcgi?db=pubmed&cmd=Retrieve&dopt=AbstractPlus&list_uids=7862109&query_hl=11&itool=pubmed_docsum) Natural vitamin D3 response elements formed by inverted palindromes: polarity-directed ligand sensitivity of vitamin D3 receptor-retinoid X receptor heterodimer-mediated transactivation. *Mol Cell Biol* **15**, 1154-61 (1995).
