# Supplementary material for: The Vitamin D Receptor Is a Wnt Effector that Controls Hair Follicle Differentiation and Specifies Tumor Type in Adult Epidermis
Source: PLoS One. 2008 Jan 23;3(1):e1483. doi: 10.1371/journal.pone.0001483 (PMC2198947; doi:10.1371/journal.pone.0001483)
Supplement: Table S3 — VDR and Lef1 binding sites in the promoter regions of Krt15, PADI3 and S1003A genes. For each gene the actual sequence present in the mouse promoter is shown (real site), together with the corresponding consensus binding site and the regions in the ChIP analysis. Sequences are numbered in order, according to their relative proximity to the transcription start (number 1 being closest). DR: Direct Repeat. IP: inverted palindrome. (0.07 MB DOC) [file pone.0001483.s007.doc]

**TABLE S3**
