# Supplementary material for: The Vitamin D Receptor Is a Wnt Effector that Controls Hair Follicle Differentiation and Specifies Tumor Type in Adult Epidermis
Source: PLoS One. 2008 Jan 23;3(1):e1483. doi: 10.1371/journal.pone.0001483 (PMC2198947; doi:10.1371/journal.pone.0001483)
Supplement: Table S4 — VDR and nuclear beta-catenin expression in human skin tumours. 59 human skin tumors were stained for VDR and beta-catenin. Expression was scored as high, medium or low. In green are highlighted all tumors with elements of hair follicle differentiation; these had high levels of both VDR and nuclear beta-catenin. In red are indicated those infiltrative BCCs that had high nuclear beta-catenin and low VDR. (0.08 MB DOC) [file pone.0001483.s008.doc]

**TABLE S4**

| Case | Age | Gender | Site | Diagnosis | Histological type | Infiltrative | Nuclear  β-cat | VDR |
| --- | --- | --- | --- | --- | --- | --- | --- | --- |
| 1 | 48 | M | Nose | TF | NT | ND | High | High |
| 2 | 62 | F | Cheek | TF | NT | ND | High | High |
| 3 | 42 | M | Cheek | TF | NT | ND | Low | High |
| 4 | 49 | F | Nose | TF | NT | ND | High | High |
| 5 | 87 | M | Nose | STF | NT | ND | High | High |
| 6 | 58 | M | Nose | TE | NT | ND | High | High |
| 7 | 74 | M | Cheek | TE | NT | ND | High | High |
| 8 | 90 | F | Cheek | BCC | solid | ND | ND | High |
| 9 | 87 | F | Ear | BCC | superficial | ND | ND | High |
| 10 | 79 | M | Abdomen | BCC | Solid | ND | ND | High |
| 11 | 63 | M | Back | BCC | superficial | ND | ND | High |
| 12 | 81 | F | Cheek | BCC | solid | ND | ND | High |
| 13 | 93 | F | Cheek | BCC | solid | ND | ND | High |
| 14 | 64 | F | abdomen | BCC | superficial | ND | ND | High |
| 15 | - | F | Scalp | BCC | solid cystic | ND | Low | Low |
| 16 | 80 | F | Nose | BCC | adenoid | ND | Low | High |
| 17 | 77 | M | Cheek | BCC | basosquamous | ND | Low | High |
| 18 | 70 | M | Check | BCC | solid | ND | Low | High |
| 19 | 26 | M | Check | BCC | Solid | ND | Low | High |
| 20 | 66 | F | Check | BCC | Solid | ND | Low | High |
| 21 | 81 | F | Nose | BCC | Solid | ND | Low | High |
| 22 | 66 | F | Scalp | BCC | micronodular | ND | Low | High |
| 23 | 45 | F | Genital area | BCC | solid cystic | ND | Low | High |
| 24 | 66 | F | Check | BCC | micronodular | ND | Low | High |
| 25 | 94 | F | Mandibular | BCC | solid cystic | ND | Low | High |
| 26 | 81 | M | Check | BCC | solid+adenoid | ND | Low | High |
| 27 | 83 | M | Ear | BCC | solid+adenoid | ND | Low | High |
| 28 | 88 | M | Scalp | BCC | Solid | ND | Low | High |
| 29 | 60 | F | Ear | BCC | adenoid+solid | ND | Low | High |
| 30 | 71 | M | Back | BCC | superficial | ND | Low | High |
| 31 | 72 | F | Check | BCC | solid | ND | Low | High |
| 32 | 66 | M | Check | BCC | solid+basosquamous | ND | Low | High |
| 33 | 72 | M | Lip | BCC | adenoid | ND | Low | High |
| 34 | 73 | M | Check | BCC | solid | ND | High | Low |
| 35 | 87 | F | Abdomen | BCC | solid+adenoid | ND | High | High |
| 36 | 57 | M | Check | BCC | follicular diff. | ND | High | High |
| 37 | 68 | M | Nose | BCC | solid | ND | High | High |
| 38 | 84 | F | Cheek | BCC | solid | ND | High | High |
| 39 | 78 | M | Cheek | BCC | fibrosing | ND | High | High |
| 40 | 78 | F | Nose | BCC | solid | + | ND | High |
| 41 | 64 | M | Cheek | BCC | solid | + | ND | High |
| 42 | 84 | F | Nose | BCC | micronodular | + | Low | High |
| 43 | 68 | F | Nose | BCC | adenoid | + | Low | High |
| 44 | 36 | M | Back | BCC | solid | + | Low | High |
| 45 | 49 | F | Nose | BCC | fibrosing | + | Low | Low |
| 46 | 69 | F | Genital area | BCC | fibrosing | + | Low | Low |
| 47 | 79 | F | Cheek | BCC | solid+fibrosing | + | High | Low |
| 48 | 72 | F | Cheek | BCC | solid | + | High | Low |
| 49 | 60 | M | Nose | BCC | adenoid | + | High | Low |
| 50 | 76 | F | Check | BCC | adenoid | + | High | Low |
| 51 | 73 | F | Forehead | BCC | Solid | + | High | Low |
| 52 | 42 | F | Scalp | BCC | fibrosing | + | High | Low |
| 53 | 75 | M | Check | BCC | fibrosing | + | High | Low |
| 54 | 25 | M | Check | BCC | solid+adenoid | + | High | Low |
| 55 | - | - | - | BCC | solid+fibrosing | + | High | Low |
| 56 | 76 | F | Check | BCC | solid | + | High | Low |
| 57 | 60 | F | Chin | BCC | micronodular | + | High | Low |
| 58 | 73 | F | Nose | BCC | fibrosing | + | High | Low |
| 59 | 75 | M | Check | BCC | Solid | + | High | Low |

TF: Trichofolliculoma

STF: Sebaceous Trichofolliculoma

TE: Trichoepithelioma

BCC: Basal Cell Carcinoma

NT: No type

ND: Non detectable
