# Supplementary material for: The Vitamin D Receptor Is a Wnt Effector that Controls Hair Follicle Differentiation and Specifies Tumor Type in Adult Epidermis
Source: PLoS One. 2008 Jan 23;3(1):e1483. doi: 10.1371/journal.pone.0001483 (PMC2198947; doi:10.1371/journal.pone.0001483)
Supplement: Table S5 — Primers used for real time PCR (0.02 MB DOC) [file pone.0001483.s009.doc]

**TABLE S5**

**Primers for Real Time PCR (forward and reverse)**:

Krt15: 5’agctattgcagagaaaaaccgt 3’, 5’ggtccgtctcaggtctgtg 3’

PADI1: 5’tgtgtgcgtggtaggtgtg 3’, 5’tcgagggatcgtagaccatgt 3’

PADI3: 5’aggattgtacgtgtatctctgga 3’, 5’ggtgtcccatagacctcaaaca 3’

Tubb3: 5’tggacagtgttcggtctgg 3’, 5’cctccgtatagtgccctttgg 3’

S1003A: 5’cagtagctgccatcgtgtg 3’, 5’tactccccaaagtccacttgcg 3’

Dlx3: 5’tcgcccaagtcggaatatacc 3’, 5’ttcaccatgcgaacctcgg 3’

Gli1: 5’tcgacctgcaaaccgtaatcc3’, 5’tcctaaagaagggctcatggta3’

Krt1-1: 5’ggtgcagatagataatgccaagc3’, 5’agctcatccaagatccttcgc3’.

**Primers for ChIP:**

Keratin 15 promoter:

Region 1: 5’caatgacctggctggttcac 3’, 5’acctctgccttacctcatag3

Region 2: 5’gctcattaaagcctctgcag 3’,5’acatgggcattgggaatctg 3’

Region 3: 5’ caaagcaaggtggctgatgg 3’, 5’ccttgtcacagctgaaaccc 3.

PADI3 promoter:

Region 1: 5’tttgcatatgaacctggggc 3’, 5’taggtttgcatgaagcatcc 3’

Region 2: 5’ggggtggcaccagaaactac 3’, 5’cacaacatttgtactgcccc 3’

Region 3: 5’ttgtgtgtgctaggctctacc 3’, 5’gaggaagctcactctctctc 3’

S1003A promoter:

Region 1: 5’agagagcccaagatgtgtcc 3’, 5’ ctctattacaggcccaccgc 3’

Region 2: 5’tgttggtctactctggcccc3’, 5’gagaaacctggcttcctggc 3’

Region 3: 5’gaggttagggtaagctggtc 3’, 5’gggtgattgctgaagatttg 3’

Unrelated genomic region:

Calponin gene ORF: 5’attcaaagctagccttgagc 3’, 5’ggccacacagtgagaacttg 3’.
